# Switching from oral atypical antipsychotic monotherapy to paliperidone palmitate once-monthly in non-acute patients with schizophrenia: A prospective, open-label, interventional study

**DOI:** 10.1007/s00213-016-4445-0

**Published:** 2016-11-05

**Authors:** Andreas Schreiner, Asaf Caspi, Paul Bergmans, Pierre Cherubin, Sofia Keim, Elsa Lara, Irina Pinchuk, Daniel Schuepbach, Sajid Suleman, Ludger Hargarter

**Affiliations:** 1 Medical & Scientific Affairs, Janssen Cilag EMEA, Johnson & Johnson Platz 1, 41470 Neuss, Germany; 2 Psychiatric Ambulatory Clinic, Sheba Medical Center, Ramat Gan, Israel; 3 Biometrics and Reporting, Janssen Cilag Benelux, Tilburg, The Netherlands; 4 Medical Affairs, Janssen Cilag EMEA, Issy-les-Moulineaux, France; 5 Global Clinical Operations EMEA MAO, Janssen Cilag, Barcarena, Portugal; 6 Hospital CUF Infante Santo, Lisbon, Portugal; 7 Ukrainian Research Institute of Social and Forensic Psychiatry and Drug Abuse, Ministry of Health of Ukraine, Kiev, Ukraine; 8 Department of Psychiatry, Psychotherapy and Psychosomatics, University Hospital of Psychiatry Zurich, Zurich, Switzerland; 9 Klinikum am Weissenhof, Weinsberg, Germany; 10 South London & Maudsley NHS Foundation Trust, Ladywell Unit, University Hospital Lewisham, London, UK

**Keywords:** Functioning, Non-acute, Long-acting injectable antipsychotic therapy, Oral antipsychotic, Paliperidone palmitate, Switching, Schizophrenia, Treatment satisfaction

## Abstract

**Rationale:**

Long-acting injectable antipsychotic therapies may offer benefits over oral antipsychotics in patients with schizophrenia.

**Objective:**

This study aimed to explore the safety, tolerability, and treatment response of paliperidone palmitate once-monthly in non-acute but symptomatic adult patients switched from previously unsuccessful monotherapy with frequently used oral atypical antipsychotics.

**Methods:**

This was a post hoc analysis of a prospective, interventional, single-arm, international, multicenter, open-label, 6-month study.

**Results:**

The patients (*N* = 472) were switched to paliperidone palmitate once-monthly (PP1M) from daily oral treatment with either aripiprazole (*n* = 46), olanzapine (*n* = 87), paliperidone extended-release (*n* = 104), quetiapine (*n* = 44), or risperidone (*n* = 191). In all groups, mean Positive and Negative Syndrome Scale total (*p* < 0.0001) and Clinical Global Impression-Severity scores improved significantly (*p* = 0.0004 to *p* < 0.0001). An improvement of ≥50 % in the Positive and Negative Syndrome Scale total score was observed in 21.7 % (aripiprazole), 29.9 % (olanzapine), 29.8 % (paliperidone extended-release), 27.3 % (quetiapine), and 37.2 % (risperidone) of patients. The patients showed significant improvements in the Personal and Social Performance score (aripiprazole *p* = 0.0409, all others *p* ≤ 0.0015); Mini International Classification of Functionality, Disability and Health Rating for Activity and Participation Disorders in Psychological Illnesses total scores (all *p* < 0.01); and Treatment Satisfaction Questionnaire for Medication Global Satisfaction score (olanzapine and risperidone *p* < 0.0001, quetiapine *p* = 0.0465, paliperidone extended-release *p* = 0.0571, aripiprazole *p* = NS). Paliperidone palmitate once-monthly was well tolerated, presenting no new safety signals.

**Conclusions:**

These data illustrate that stable, non-acute but symptomatic patients on oral antipsychotic monotherapy may show clinically meaningful improvement of symptoms, functioning, and treatment satisfaction after direct transition to PP1M. The findings are limited by the naturalistic study design; thus, further studies are required to confirm the current findings.

**Electronic supplementary material:**

The online version of this article (doi:10.1007/s00213-016-4445-0) contains supplementary material, which is available to authorized users.

## Introduction

Pharmacotherapy, which includes oral and long-acting injectable (LAI) antipsychotics, remains the mainstay treatment in schizophrenia (Hasan et al. 2013); nevertheless, discontinuation rates are high (Kahn et al. 2008; Lieberman et al. 2005; Naber and Lambert 2009). Clinical practice guidelines strongly recommended antipsychotic monotherapy for the treatment of schizophrenia (Barnes 2011; Lerma-Carrillo et al. 2008; National Institute for Health and Care Excellence (NICE) 2014).

Oral atypical antipsychotics generally have the same overall efficacy but differ in their side effect profiles, with apparent differences in sedation, metabolic disturbances such as weight gain, glucose and lipid abnormalities, and the risk of extrapyramidal motor symptoms (EPMSs) (Davis et al. 2003; De Hert et al. 2012; Jones et al. 2010; Leucht et al. 2013; Rummel-Kluge et al. 2010). Non-adherence to antipsychotic medication is associated with increased risk of relapse and hospitalization at all stages of schizophrenia (Kozma and Weiden 2009; Leucht and Heres 2006; Llorca 2008; Robinson et al. 1999), and may be influenced by several factors (Tandon et al. 2006), including poor efficacy (worsening of symptoms) (Liu-Seifert et al. 2005) and the presence of side effects (Higashi et al. 2013).

LAI antipsychotic therapy (LAT) may improve adherence to medication in patients with schizophrenia (Cañas et al. 2013) and, as a consequence, significantly reduce relapse rates and improve long-term outcomes compared with those treated with oral antipsychotic medication (Leucht et al. 2011; Tiihonen et al. 2011). A systematic review and meta-analysis of mirror-image studies comparing a period of treatment using oral antipsychotics with a subsequent period of treatment using LAT within the same patient showed that LATs were significantly superior in reducing relapse rates compared with oral antipsychotics (Kishimoto et al. 2013); however, this outcome was in contrast to a recent meta-analysis based on randomized controlled trials (RCTs) comparing LATs and oral antipsychotics (Kishimoto et al. 2014). The short duration of the majority of RCTs, the very defined patient inclusion criteria, and the added care and close follow-up of patients suggest that such studies do not adequately reflect real clinical practice or treatment of patients with schizophrenia in the community, and therefore longer, naturalistic studies are required (Olivares et al. 2009b). Consequently, it has been shown that the outcomes of studies comparing oral antipsychotics and LATs are sensitive to trial design, and RCTs are not optimal for exploring differences between oral antipsychotics and LATs (Alphs et al. 2014; Kirson et al. 2013).

Since treatment response and side effect profiles vary between different antipsychotics (Jones et al. 2010; Leucht et al. 2013; Taylor et al. 2012), it is of particular interest to explore treatment response, safety, and tolerability in patients previously unsuccessfully treated with oral atypical antipsychotic monotherapy who were switched to LAT in a routine clinical setting. Paliperidone palmitate once-monthly (PP1M) is an atypical LAT designed for intramuscular (IM) administration indicated for the maintenance treatment of adult patients with schizophrenia (Janssen Cilag 2015).

The Paliperidone Palmitate Flexible Dosing in Schizophrenia (PALMFlexS) study was a prospective, 6-month, pragmatic, interventional study conducted in a large, more representative sample of patients with schizophrenia than those recruited in the pivotal RCTs (Hargarter et al. 2015; Schreiner et al. 2015b; Schreiner et al. 2014a). The study was designed specifically to reflect more closely real-world clinical situations in which the transition to another antipsychotic is performed in previously unsuccessfully treated patients. The PALMFlexS study included three distinct patient populations: patients with non-acute schizophrenia switching to PP1M from oral antipsychotics, non-acute patients switching to PP1M from other LATs, and acute patients switching to PP1M from oral antipsychotics.

To understand the impact of dosing and switching strategies when initiating PP1M, the current analysis was conducted in non-acute but symptomatic patients with schizophrenia switched from previously unsuccessful monotherapy with the most frequently used oral atypical antipsychotics.

## Materials and methods

This was a post hoc analysis of a prospective, interventional, single-arm, multicenter, open-label, 6-month study performed in patients with schizophrenia from 160 centers across 21 countries between November 2010 and November 2012 (NCT01281527). The study was performed in accordance with the Declaration of Helsinki and was consistent with Good Clinical Practices of the International Conference on Harmonisation and applicable regulatory requirements. All the patients provided informed written consent.

The protocol, full details of the study population, and overall results have previously been reported (Schreiner et al. 2014a). Methods that are specific to this post hoc analysis are briefly described below.

### Study design and patients

Non-acute but symptomatic male or female patients aged ≥18 years with schizophrenia (diagnosed according to the *Diagnostic and Statistical Manual of Mental Disorders*, *Fourth Edition* criteria), who were previously unsuccessfully treated using oral monotherapy with either aripiprazole (ARI), olanzapine (OLA), paliperidone extended-release (Pali ER), quetiapine (QUE), or risperidone (RIS), were selected from the overall study population of PALMFlexS (Schreiner et al. 2014a).

The patients were required to be “stable” but symptomatic, i.e., have been on the same oral atypical antipsychotic monotherapy for the treatment of schizophrenia on an adequate therapeutic dose and with a change in the Clinical Global Impression-Severity (CGI-S) score of ≤1 for ≥4 weeks prior to enrolment. Their current treatment was considered to have been unsuccessful due to one or more of the following reasons: lack of efficacy (baseline Positive and Negative Syndrome Scale [PANSS] total score ≥70 or ≥2 items scoring ≥4 in the PANSS positive or negative subscale or ≥3 items scoring ≥4 in the PANSS general psychopathology subscale, as judged by the investigator), lack of tolerability or safety (defined as the presence of intolerable [according to the patient] and/or clinically relevant [according to the investigator] side effects on their current antipsychotic medication), lack of adherence, or patient’s wish. Lack of adherence was assessed individually by the investigator. There were no specific protocol-defined criteria. Patients were excluded if, at the discretion of the investigator, their diagnosis was considered to be the direct result of the pharmacological effects of a substance or general medical condition, they were treatment naïve, they had received clozapine within 3 months prior to the start of the study, they were considered at imminent risk of suicide even after clinical intervention, they had a history of or current symptoms of tardive dyskinesia or neuroleptic malignant syndrome, they were pregnant or breastfeeding, or they had any known allergies to RIS or paliperidone or any of its excipients. The inclusion/exclusion criteria were designed to recruit a more diverse study population than those in previously conducted pivotal studies (Gopal et al. 2010; Pandina et al. 2010); for example, patients with relevant comorbidities, co-medications, and current substance use or abuse, with the exception of intravenous drug use, were eligible for enrolment and there were no exclusions based on body mass index (BMI).

In the present study, the patients were switched directly to PP1M, in line with the indication and posology of PP1M European summary of product characteristics (Janssen Cilag 2015). After initiation of PP1M, the patients were tapered off their oral atypical antipsychotic at the discretion of the treating physician, preferably within a maximum of 4 weeks. PP1M was initiated, in line with the SmPC, at a recommended dose of 150 mg equivalent (mg eq) on day 1 and 100 mg eq on day 8 (±2 days; nota bene, the current summary of product characteristics now states ±4 days) intramuscularly, both given in the deltoid muscle. Subsequently, PP1M was administered once-monthly (±7 days) (visit days) using flexible maintenance dosages within the range of 50 to 150 mg eq based on the clinical judgment of the treating physician. Patients without documentation of previous RIS or paliperidone exposure were tested for tolerability with Pali ER (3 mg/day) for at least 2 days prior to receiving PP1M. Efficacy, tolerability, and safety were assessed by the same person at each study visit, whenever possible.

### Efficacy assessments

Efficacy outcomes were assessed after 6 months of treatment by trained, qualified, non-blinded assessors. Evaluation time points were days 1 (baseline), 8, 38 (month 1), 68 (month 2), 98 (month 3), 128 (month 4), 158 (month 5), and 188 (month 6). The primary efficacy outcome for non-acute but symptomatic patients with schizophrenia switched due to lack of efficacy was the percentage of patients achieving treatment response, defined as ≥20 % improvement in PANSS total score from baseline to endpoint (6 months or time of early discontinuation). Maintained efficacy (defined as non-inferiority in the change in PANSS total score at endpoint versus baseline, as measured by means of Schuirmann’s test) was the primary efficacy outcome for patients switched to PP1M for other reasons. Actual scores and change from baseline in CGI-S score, Personal and Social Performance (PSP) scale (Morosini et al. 2000) total score, Mini International Classification of Functionality, Disability and Health (ICF) Rating for Activity and Participation Disorders in Psychological Illnesses (Mini-ICF-APP) (Linden and Baron 2005; Molodynski et al. 2013), and treatment satisfaction (assessed in patients using the 14-item Treatment Satisfaction Questionnaire for Medication [TSQM] scale (Atkinson et al. 2004) and physician treatment satisfaction using a 7-point categorical scale) scores were also analyzed.

### Safety and tolerability

All treatment-emergent adverse events (TEAEs), defined as adverse events that were new in onset or were aggravated in severity following initiation of PP1M, were documented at each clinic visit and coded using the Medical Dictionary for Regulatory Activities (version 13.0). EPMSs were assessed by the Extrapyramidal Symptom Rating Scale (ESRS) (Chouinard and Margolese 2005). In addition, alcohol and substance use were measured using the Clinician Rating Alcohol Use Scale (CRAUS) and the Clinician Rating Substance Use Scale (CRSUS) (Carey et al. 1996). Body weight was recorded at each assessment point and endpoint, and BMI was calculated. There were no obligatory protocol-based prolactin measurements; however, investigators were allowed to measure prolactin levels at any time during the study at their own discretion.

### Data analysis

The intent-to-treat (ITT) population comprised all patients who received PP1M at least once. Analysis of treatment response was performed on the efficacy analysis population, which included all ITT patients with at least one post-baseline efficacy assessment. Endpoint analysis using the last observation carried forward (LOCF) method was performed in addition to observed case analysis. Actual values and changes from baseline were summarized descriptively at each assessment time point and at the patient’s last evaluation (endpoint) while categorical variables were summarized with frequency and percentage. Within-group changes in efficacy parameters from baseline to endpoint were analyzed using the Wilcoxon signed-rank test. Between-oral-subgroup differences were tested using Fisher’s exact test and the Kruskal–Wallis test. All tests were performed using Statistical Analysis System version 9.2.

Safety and tolerability were evaluated throughout the study on the safety ITT population, which comprised all ITT patients who had at least one post-baseline safety observation. TEAE frequency distributions included severity of events (i.e., mild, moderate, or severe) and causal relationship to treatment (i.e., not related, doubtful, possible, probably, or very likely).

## Results

### Demographics and patient disposition

In total, 472 non-acute patients with schizophrenia were eligible for this analysis. Patients enrolled in the study were on a stable oral atypical antipsychotic dose at enrolment (Table 1). The reasons for patients to switch from their current oral atypical antipsychotic to PP1M were patient’s wish (45 %), lack of efficacy (22 %), and lack of compliance (25 %).

**Table 1:** Baseline characteristics and PP1M dosing (*N* = 472)

Patients switched to PP1M from	ARI	OLA	Pali ER	QUE	RIS	*p* value
ITT population, *n*	46	87	104	44	191	
Mean age, years (SD)	34.4 (9.4)	36.8 (11.6)	37.7 (11.7)	40.8 (11.7)	38.7 (12.5)	0.0773^a^
Male, %	73.9	67.8	70.2	50.0	61.8	0.0861^b^
Mean age at diagnosis, years (SD)	27.6 (6.7)	28.4 (9.7)	29.0 (9.5)	30.7 (10.3)	30.7 (10.4)	
Diagnosis of paranoid schizophrenia, %	78.3	79.3	81.7	72.7	74.9	
Mean baseline weight, kg (SD)	89.1 (22.1)	79.8 (16.4)	80.0 (17.3)	80.4 (16.0)	79.7 (17.3)	0.1018^a^
Mean baseline BMI, kg/m^2^ (SD; range)	29.9 (7.5; 17, 51)	27.2 (6.1; 18, 46)	27.0 (5.5; 17, 51)	28.1 (5.3; 18, 39)	27.0 (5.5; 17, 54)	0.1120^a^
Patients with ≥1 comorbidity, %^c^	78.3	63.2	58.7	52.3	59.2	
Number of previous hospitalizations, %						
None	10.9	18.4	21.2	18.2	19.9	
1–3	45.7	47.1	35.6	36.4	48.7	
≥4	43.5	34.5	43.3	45.5	31.4	
Patients with diagnosed substance abuse (with or without impairment), %	9.8	10.5	6.4	5.4	11.1	
Mean daily dose of prior antipsychotic, mg (SD)	22.7 (10.7)	15.6 (8.2)	7.6 (2.6)	482.4 (277.1)	4.3 (2.3)	
Patients receiving PP1M initiation regimen at day 1 and day 8 according to label, %^d^	93.5	95.4	92.3	97.7	96.3	
Mean modal PP1M maintenance dose, mg eq (SD)^e^	94.9 (35.0)	104.2 (33.6)	100.5 (32.3)	105.0 (36.8)	98.9 (32.3)	
Last PP1M dose received, % of patients						
50 mg eq	19.6	6.9	8.7	13.6	8.4	
75 mg eq	19.6	32.2	32.7	15.9	35.1	
100 mg eq	30.4	31.0	35.6	40.9	33.0	
150 mg eq	30.4	29.9	23.1	29.5	23.6	
Relevant co-medications						
Number (%) of patients using benzodiazepines						
At baseline	12 (26.1)	25 (28.7)	28 (26.9)	9 (20.5)	39 (20.4)	
Newly initiated during study	11 (23.9)	21 (24.1)	26 (25.0)	14 (31.8)	30 (15.7)	
At endpoint	12 (26.1)	21 (24.1)	21 (20.2)	8 (18.2)	36 (18.8)	
At 6 months for completers^f^	9 (29.0)	10 (17.5)	15 (18.1)	6 (18.8)	29 (18.2)	
Number (%) of patients using anticholinergics						
At baseline	2 (4.3)	8 (9.2)	10 (9.6)	4 (9.1)	23 (12.0)	
Newly initiated during study	2 (4.3)	9 (10.3)	7 (6.7)	4 (9.1)	14 (7.3)	
At endpoint	2 (4.3)	8 (9.2)	5 (4.8)	4 (9.1)	14 (7.3)	
At 6 months for completers^f^	2 (6.5)	5 (8.8)	4 (4.8)	2 (6.3)	12 (7.5)	

*ARI* aripiprazole, *BMI* body mass index, *ITT* intent to treat, *OLA* olanzapine, *Pali ER* paliperidone extended-release, *PP1M* once-monthly paliperidone palmitate, *QUE* quetiapine, *RIS* risperidone, *SD* standard deviation. *p* values indicate differences between prior oral antipsychotic treatment subgroups

^a^Kruskal–Wallis test

^b^Fisher’s exact test

^c^Individual patients can be labelled for >1 comorbidity

^d^The recommended initiation regimen was PP1M 150 mg eq on day 1 and 100 mg eq on day 8, given in the deltoid muscle

^e^Excluding the initiation regimen (day 1/day 8)

^f^ARI *n* = 31; OLA *n* = 57; Pali ER *n* = 83; QUE *n* = 32; RIS *n* = 159

**Fig. 1:**
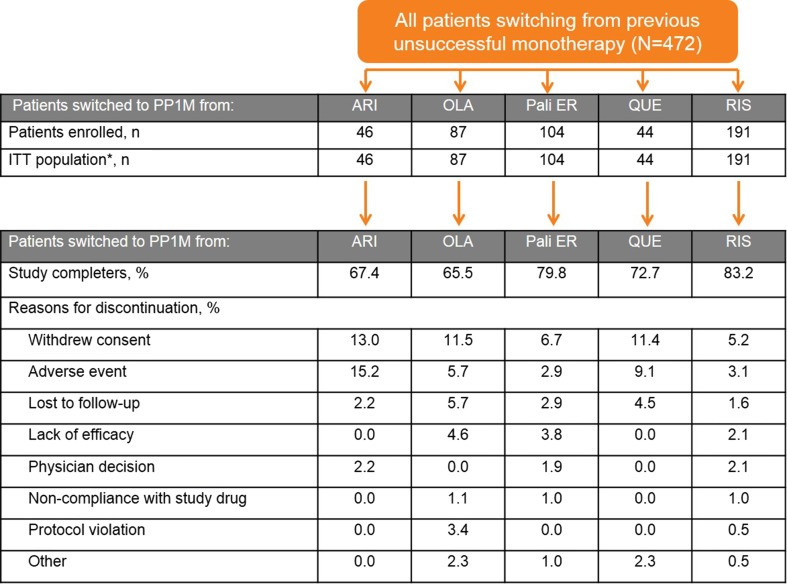
Patient disposition. *patients who received at least one dose of study drug. *ARI* aripiprazole, *ITT* intent to treat, *OLA* olanzapine, *PP1M* once-monthly paliperidone palmitate, *Pali ER* paliperidone extended-release, *QUE* quetiapine, *RIS* risperidone

Patient disposition is described in Fig. 1. Baseline characteristics are summarized in Table 1. There were non-significant between-group differences in mean age (standard deviation [SD]; ranging from 34.4 [9.4] years [ARI] to 40.8 [11.7] years [QUE]), mean body weight (79.7 [17.3] kg [RIS] to 89.1 [22.1] kg [ARI]), and percentage of males in the group (50.0 % [QUE] to 73.9 % [ARI]) at baseline (Table 1). At baseline, 39 (9.3 %) patients were reported to have a diagnosis of substance abuse (with or without impairment).

Following the day 1 (150 mg eq)/day 8 (100 mg eq) initiation regimen, the PP1M mean modal maintenance dose from the third injection onwards ranged from 94.9 (35.0) mg eq for patients who switched from ARI to 105.0 (36.8) mg eq for patients who switched from QUE; the final dose distribution of PP1M is summarized in Table 1. Regardless of the oral antipsychotic that patients were switched from, most (92.3 % [Pali ER] to 97.7 % [QUE]) received PP1M according to the recommended initiation regimen. Overall, 72.8 % of patients had a dose adjustment after the third dose of PP1M, with the majority requiring only one adjustment (ARI 45.7 %, OLA 54.0 %, Pali ER 49.0 %, QUE 45.5 %, RIS 50.8 %). The percentage of patients with one or more dose increase varied from 36.5 % (Pali ER) to 45.5 % (QUE), and the percentage of patients with one or more dose decrease varied from 38.6 % (QUE) to 55.8 % (Pali ER).

The proportion of patients using concomitant medications (benzodiazepines and anticholinergics) during the study is shown in Table 1.

### Efficacy outcomes

**Fig. 2:**
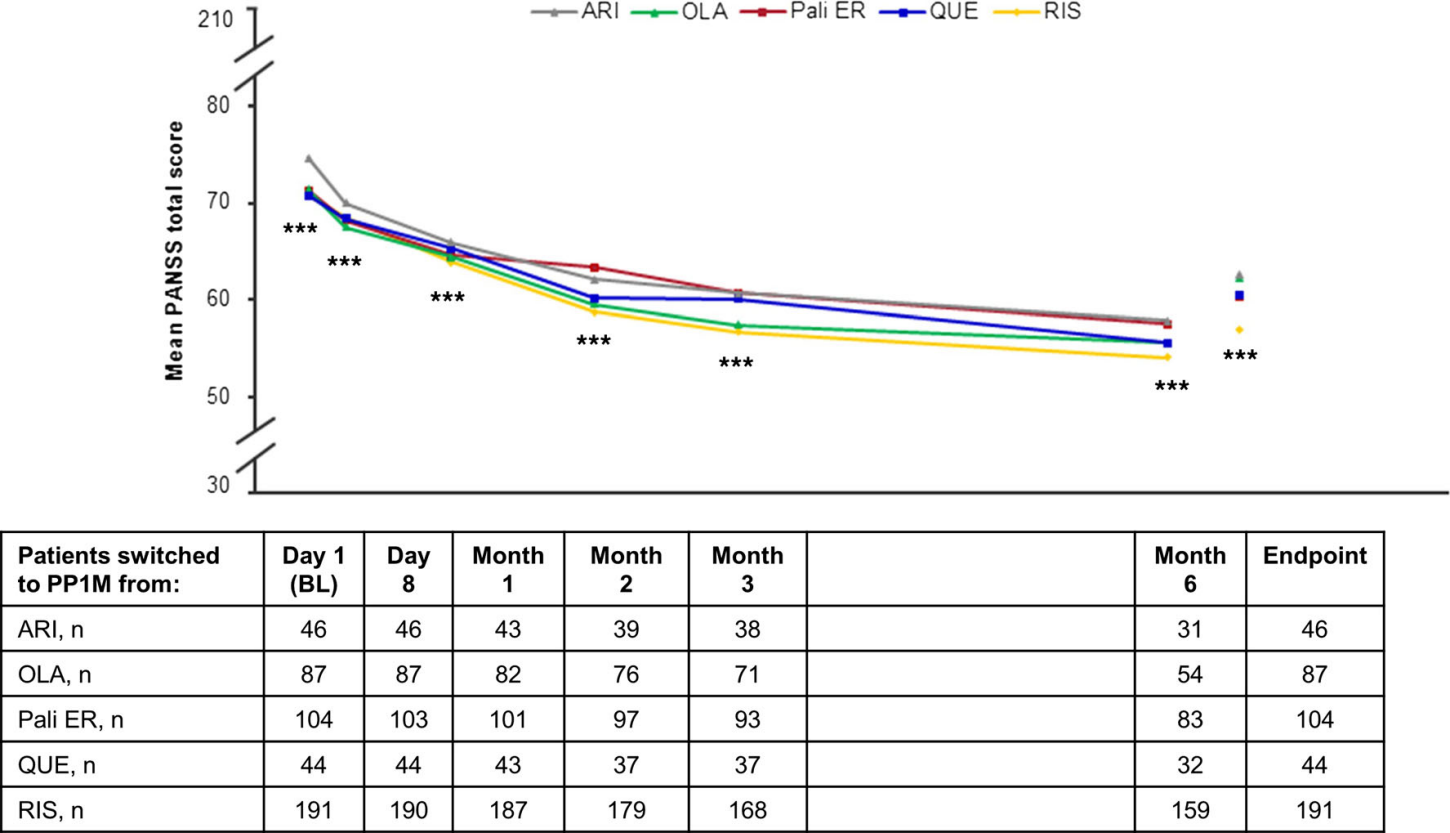
Mean PANSS total score over time (efficacy ITT population; *N* = 472). *p* < 0.001 vs baseline for all antipsychotics. *ARI* aripiprazole, *BL* baseline, *ITT* intent to treat, *OLA* olanzapine, *PANSS* Positive and Negative Syndrome Scale, *PP1M* once-monthly paliperidone palmitate, *Pali ER* paliperidone extended-release, *QUE* quetiapine, *RIS* risperidone. ***p <0.0001 vs baseline for all antipsychotics

At endpoint, of all patients that switched from previous atypical oral monotherapy to PP1M, 52.2 % (ARI), 60.9 % (OLA), 57.7 % (Pali ER), 65.9 % (QUE), and 73.8 % (RIS) had a ≥20 % improvement in PANSS total score. In addition, 21.7 % (ARI), 29.9 % (OLA), 29.8 % (Pali ER), 27.3 % (QUE), and 37.2 % (RIS) of patients had a ≥50 % improvement in PANSS total score. The mean PANSS total score was significantly improved from baseline to endpoint in all groups (*p* < 0.0001) (Fig. 2). Disease severity, as measured by the mean CGI-S score, improved significantly from baseline to endpoint in all groups (*p* = 0.0004 to *p* < 0.0001) (Table 2).

**Table 2:** Secondary efficacy outcomes (efficacy ITT population; *N* = 472)

Patients switched to PP1M from	ARI	OLA	Pali ER	QUE	RIS
Mean PANSS total score, *n*	44	87	104	44	191
Baseline (SD)	74.7 (14.9)	71.4 (13.2)	71.3 (14.3)	70.8 (13.1)	70.8 (15.1)
Endpoint (SD)	62.6 (16.5)	62.3 (19.6)	60.4 (17.2)	60.5 (20.1)	56.9 (17.3)
Mean change from baseline to endpoint (SD)	−12.2 (16.7)	−9.1 (17.5)	−10.8 (14.4)	−10.2 (19.6)	−13.9 (14.8)
95 % CI of mean change (*p* value^b^)	−17.1, −7.2 (< 0.0001)	−12.9, −5.4 (< 0.0001)	−13.6, −8.0 (< 0.0001)	−16.2, −4.3 (< 0.0001)	−16.1, −11.8 (< 0.0001)
Mean CGI-S score, *n* ^a^	46	86	104	44	189
Baseline (SD)	4.1 (0.8)	3.7 (1.0)	3.9 (0.9)	3.9 (0.9)	3.8 (0.9)
Endpoint (SD)	3.5 (1.0)	3.3 (1.2)	3.4 (1.1)	3.4 (1.0)	3.0 (1.0)
Mean change from baseline to endpoint (SD)	−0.6 (1.1)	−0.4 (1.1)	−0.6 (1.1)	−0.5 (1.1)	−0.8 (0.9)
95 % CI of mean change (*p* value^b^)	−0.9, −0.3 (0.0003)	−0.6, −0.2 (0.0003)	−0.8, −0.4 (<0.0001)	−0.9, −0.2 (0.0004)	−0.9, −0.7 (<0.0001)
Mean PSP score, *n* ^a^	44	84	103	44	185
Baseline (SD)	58.9 (13.4)	61.5 (14.6)	58.3 (13.7)	56.3 (12.0)	57.8 (12.3)
Endpoint (SD)	62.9 (15.2)	66.0 (17.7)	65.4 (16.4)	64.2 (15.9)	68.2 (13.9)
Mean change from baseline to endpoint (SD)	3.9 (13.2)	4.5 (15.9)	7.0 (13.8)	7.9 (12.4)	10.4 (13.8)
95 % CI of mean change (*p* value^b^)	−0.1, 8.0 (0.0409)	1.1, 8.0 (0.0015)	4.3, 9.7 (<0.0001)	4.1, 11.6 (<0.0001)	8.4, 12.4 (<0.0001)
Mean Mini-ICF-APP total score, *n* ^c^	43	79	97	42	179
Baseline (SD)	19.0 (7.8)	18.1 (8.8)	19.9 (8.5)	21.6 (6.9)	19.9 (7.0)
Endpoint (SD)	16.1 (9.8)	15.3 (9.5)	16.8 (9.6)	17.8 (8.7)	14.7 (7.3)
Mean change from baseline to endpoint (SD)	−2.9 (7.1)	−2.8 (7.8)	−3.1 (7.3)	−3.8 (9.2)	−5.2 (7.1)
95 % CI of mean change (*p* value^b^)	−5.1, −0.7 (0.0079)	−4.5, −1.1 (0.0013)	−4.5, −1.6 (<0.0001)	−6.7, −0.9 (0.0015)	−6.3, −4.2 (<0.0001)
Physician overall treatment satisfaction, *n*	41	76	94	38	172
Baseline (SD)	4.5 (1.1)	4.1 (1.2)	3.1 (1.0)	4.1 (1.1)	3.9 (1.1)
Endpoint (SD)	2.8 (1.1)	2.7 (1.2)	2.4 (1.0)	2.7 (1.3)	2.2 (0.9)
Mean change from baseline to endpoint (SD)	−1.7 (1.5)	−1.4 (1.6)	−0.7 (1.3)	−1.4 (1.6)	−1.7 (1.4)
95 % CI of mean change (*p* value^b^)	−2.1, −1.2 (<0.0001)	−1.7, −1.0 (<0.0001)	−0.9, −0.4 (<0.0001)	−1.9, −0.9 (<0.0001)	−1.9, −1.5 (<0.0001)
Patient global treatment satisfaction (TSQM), *n*	35	73	90	34	166
Baseline (SD)	58.8 (22.1)	55.5 (20.8)	60.2 (22.8)	54.2 (21.7)	54.1 (19.5)
Endpoint (SD)	57.6 (25.2)	68.6 (24.4)	64.9 (22.5)	63.7 (24.2)	67.1 (24.4)
Mean change from baseline to endpoint (SD)	−1.2 (32.2)	13.1 (26.0)	4.8 (24.7)	9.5 (28.9)	13.1 (30.0)
95 % CI of mean change (*p* value^b^)	−12.3, 9.8 (0.7593)	7.1, 19.2 (<0.0001)	−0.4, 9.9 (0.0571)	−0.6, 19.5 (0.0465)	8.5, 17.7 (<0.0001)

Only patients with a valid baseline measurement and at least one valid follow-up assessment were included

*ARI* aripiprazole; *CGI-S* Clinical Global Impression-Severity; *CI* confidence interval; *ITT* intent to treat; *Mini-ICF-APP* Mini International Classification of Functionality, Disability and Health Rating for Activity and Participation Disorders in Psychological Illnesses; *OLA* olanzapine; *Pali ER* paliperidone extended-release; *PANSS* Positive and Negative Syndrome Scale; *PP1M* once-monthly paliperidone palmitate; *PSP* Personal and Social Performance; *QUE* quetiapine; *RIS* risperidone; *SD* standard deviation; *TSQM* Treatment Satisfaction Questionnaire for Medication

^a^For the CGI-S a lower score indicates improvement; For the PSP, a higher score indicates improvement

^b^Within-group difference was tested using the Wilcoxon signed-rank test

^c^For the Mini-ICF-APP, a lower score indicates improvement

### Secondary outcomes

At endpoint, patients who were switched from atypical oral antipsychotics to PP1M showed a statistically significant improvement from baseline in PSP total scores (*p* = 0.0409 for ARI to *p* < 0.0001 for RIS, Pali ER, and QUE) and in Mini-ICF-APP total score (*p* = 0.0079 for ARI to *p* < 0.0001 for RIS and Pali ER) (Table 2). Significant improvements from baseline to endpoint in TSQM Global Satisfaction score were observed in patients who switched from OLA (*p* < 0.0001), QUE (*p* = 0.0465), and RIS (*p* < 0.0001) and in patients who switched from Pali ER (*p* = 0.0571). There was no significant difference in TSQM Global Satisfaction score between ARI and PP1M. Overall, physician treatment satisfaction improved significantly from baseline to endpoint in all groups (*p* < 0.0001) (Table 2).

### Safety and tolerability

**Fig. 3:**
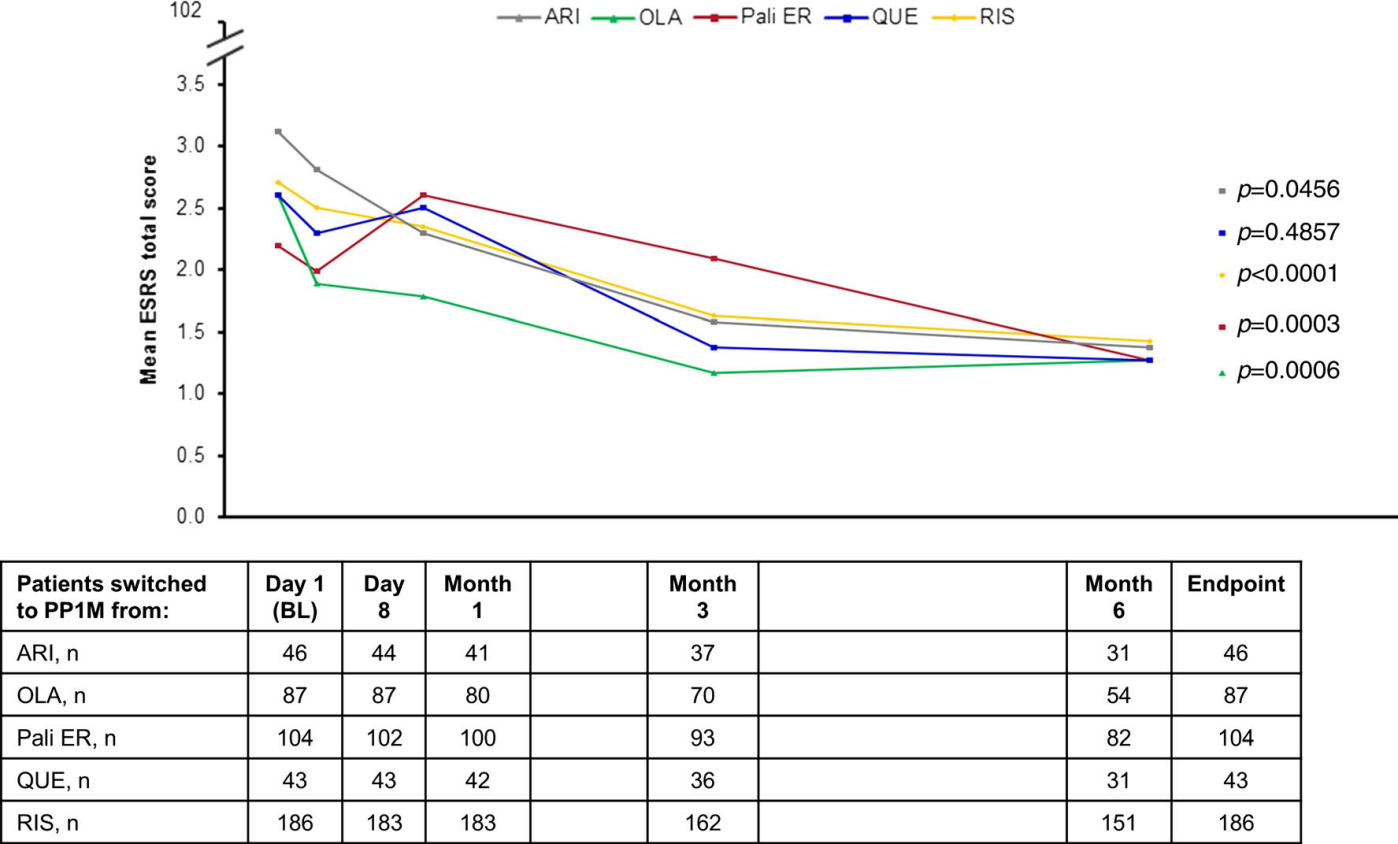
Mean ESRS total score over time (safety ITT population; *N* = 472). *ARI* aripiprazole, *BL* baseline, *ESRS* Extrapyramidal Symptom Rating Scale, *ITT* intent to treat, *OLA* olanzapine, *PP1M* once-monthly paliperidone palmitate, *Pali ER* paliperidone extended-release, *QUE* quetiapine, *RIS* risperidone *p* values represent are for change from baseline to endpoint, Wilcoxon-signed-rank test

TEAEs affecting ≥5 % of patients in any group are summarized in Table 3. Mean change in the ESRS total score (SD) from baseline to endpoint was −0.6 (3.4) for ARI, −1.3 (4.4) for OLA, −0.7 (4.1) for Pali ER, −0.3 (3.2) for QUE, and −1.2 (3.5) for RIS (*p* < 0.05 for all, except QUE *p* = 0.4857) (Fig. 3). Mean weight change (SD) from baseline to endpoint ranged between −0.3 (4.6) kg for OLA (95 % confidence interval [CI] –1.3, 0.7) and 3.5 (6.3) kg for ARI (95 % CI 1.5, 5.4) (Supplementary Table 1). Mean changes in BMI from baseline to endpoint were similar, ranging from −0.1 (1.6) kg/m^2^ for OLA (95 % CI −0.5, 0.3) to 1.2 (2.2) kg/m^2^ for ARI (95 % CI 0.5, 1.9) (Supplementary Table 1).

**Table 3:** Treatment-emergent adverse events (safety ITT population; *N* = 472)

Patients switched to PP1M from	ARI (*n* = 46)	OLA (*n* = 87)	Pali ER (*n* = 104)	QUE (*n* = 44)	RIS (*n* = 191)
Total number of TEAEs^a^	135	178	158	104	241
Mild (%)	74 (54.8)	112 (62.9)	106 (67.1)	53 (51.0)	134 (55.6)
Moderate (%)	52 (38.5)	56 (31.5)	43 (27.2)	46 (44.2)	87 (36.1)
Severe (%)	9 (6.7)	10 (5.6)	9 (5.7)	5 (4.8)	20 (8.3)
Subjects with ≥1 TEAE^a^, *n* (%)	24 (52.2)	45 (51.7)	39 (37.5)	19 (43.2)	53 (27.7)
TEAEs^a^ occurring in ≥5 % of patients in any group, *n* (%)					
Injection-site pain	3 (6.5)	12 (13.8)	15 (14.4)	7 (15.9)	16 (8.4)
Akathisia	4 (8.7)	5 (5.7)	5 (4.8)	2 (4.5)	5 (2.6)
Somnolence	2 (4.3)	5 (5.7)	2 (1.9)	4 (9.1)	4 (2.1)
Abnormal weight gain^b^	3 (6.5)	1 (1.1)	0 (0.0)	2 (4.5)	3 (1.6)
Weight increased^b^	3 (6.5)	1 (1.1)	3 (2.9)	4 (9.1)	3 (1.6)
Insomnia	0 (0.0)	7 (8.0)	1 (1.0)	2 (4.5)	2 (1.0)
Psychotic disorders	1 (2.2)	6 (6.9)	4 (3.8)	1 (2.3)	1 (0.5)

*ARI* aripiprazole, *ITT* intent to treat, *MedDRA* Medical Dictionary for Regulatory Activities, *OLA* olanzapine, *Pali ER* paliperidone extended-release, *PP1M* once-monthly paliperidone palmitate, *QUE* quetiapine, *RIS* risperidone, *TEAE* treatment-emergent adverse event

^a^Possibly, probably, or very likely related to PP1M treatment

^b^Based on the MedDRA coding system, both terms were applicable for coding of TEAEs. None of the identified subjects were recorded under both terms simultaneously

Investigators reported hyperprolactinemia in two patients in the OLA group and three patients in the prior Pali ER group and an increase in blood prolactin in two patients in the prior ARI group.

Of 1058 PALMFlexS patients, 177 did not have source documentation on PP1M/RIS exposure. A total 172 patients tolerated the oral tolerance test well, two patients did not, and in three patients, the test result was not available.

## Discussion

In this analysis, patients with non-acute schizophrenia who were switched from oral atypical antipsychotic monotherapy to PP1M demonstrated significant and clinically relevant improvements in psychotic symptoms and functioning, regardless of the previous oral atypical antipsychotic monotherapy.

At baseline, patients were mildly to moderately symptomatic, and as such, they could be considered stable but sub-optimally controlled. After 6 months of treatment with PP1M, over half of all patients showed a treatment response, defined a priori as a reduction in total PANSS score of ≥20 %, which represents a clinically meaningful improvement (Cook et al. 2002) for non-acute patients. Improved efficacy of ≥20 % was specified as the primary endpoint in this subgroup of patients, as they were considered previously stable by their treating physician for at least 1 month prior to enrolment while being prescribed an adequate dose of an oral atypical antipsychotic monotherapy. Therefore, the improvement would not be expected to be comparable with what generally would be observed in acutely ill patients, where ≥30 or ≥50 % improvements in PANSS total score are considered more adequate (Leucht 2014; Leucht et al. 2005). Nevertheless, a ≥50 % reduction in PANSS total score was achieved by approximately one quarter of patients. The present results are consistent with a recent naturalistic study where patients previously unsuccessfully treated with oral antipsychotic medications were switched to an atypical LAT (RIS) and showed significant improvements in hospitalization days and psychotic symptoms (Schreiner et al. 2014b). However, between 17 and 32 % of patients who were unsuccessfully treated with other LAI antipsychotics achieved a ≥50 % improvement in their PANSS total score after switching to PP1M (Schreiner et al. 2015b).

PP1M maintenance doses were relatively homogeneously distributed across the groups independent of the previous oral antipsychotic and in line with those expected based on the prior mean oral doses. Exceptions were the higher maintenance doses following a switch from QUE, possibly to compensate for the loss of sedating effects and the lower maintenance doses in patients with prior ARI use, who represented the youngest patients and those with the shortest time since diagnosis, who were therefore likely to require relatively lower antipsychotic doses.

Differential outcomes were observed in efficacy, functioning, EPMS improvement, and weight gain, depending on the previous oral antipsychotic monotherapy received prior to switching to PP1M and consistent with the heterogeneity existing within the group of atypical antipsychotics. When patients were assessed according to previous oral antipsychotic monotherapy, improvements in efficacy and functioning scores were numerically greater for those patients who switched to PP1M from RIS than from other oral antipsychotics. With regard to EPMS, patients from all groups other than QUE experienced significant improvements, with the greatest improvements observed for those patients switching from oral RIS and OLA. Patients switching to PP1M from previous oral monotherapy with QUE and ARI showed greater weight gain compared with other oral antipsychotic switch groups. However, caution should be exercised when interpreting these differences as these were exploratory analyses only and the choice of the previous oral antipsychotic medication actually may have been influenced by the propensity of a patient to develop weight gain or EPMS, which would be supported by the observation that patients previously treated with oral ARI had the highest baseline body weight and BMI.

The current analysis supports the results from previous fixed-dose RCTs and confirms the efficacy of PP1M in the treatment of schizophrenia (Gopal et al. 2010; Pandina et al. 2010), and further expands on results from retrospective and prospective studies in stabilized patients with schizophrenia that demonstrated improvements in clinical symptoms and functioning when switched from an oral to a long-acting antipsychotic medication (Möller et al. 2005; Olivares et al. 2009a; Rosa et al. 2012), as well as from a LAI antipsychotic to PP1M (Schreiner et al. 2015b).

Poor adherence to antipsychotic medication impacts the management of schizophrenia; therefore, strategies aimed at improving treatment adherence are important to achieve optimal long-term clinical outcomes (Cañas et al. 2013). LAI antipsychotics offer a number of potential benefits in long-term maintenance treatment of patients with schizophrenia, providing assured delivery of medication, regular contact with the healthcare team, and transparency about the treatment schedule, as healthcare professionals will immediately be aware if a patient misses a dose appointment (Cañas et al. 2013). A recent Italian survey reported that psychiatrists consider that switching patients to a LAI antipsychotic is a suitable approach to improve adherence in patients who are not optimally controlled with an oral atypical antipsychotic (de Bartolomeis et al. 2016). In this context, the findings from the current analysis provide evidence of the potential impact of switching to LAT with paliperidone palmitate in a real-world setting with a heterogeneous patient population representative of clinical practice.

Naturalistic studies such as this one reflect everyday clinical settings more accurately than RCTs but are subject to a number of limitations that should be kept in mind when interpreting the findings. The study was not designed to detect differential effects of previous oral antipsychotic monotherapy on patient outcomes, and therefore, the post hoc data presented herein with respect to such data should be considered exploratory in nature. Instead, a prospective pragmatic study design would enable analyses of the effectiveness and safety of PP1M when switching from different oral antipsychotic monotherapies. The study was open-label with no active comparator group, and as such, these data do not provide a head-to-head comparison between treatments; rather, they suggest that failure with one antipsychotic medication does not predict failure with another. Increased time spent in the study and with healthcare professionals may have added to the observed improvement in outcomes.

Other limitations associated with an open-label design also impact the interpretation and generalizability of the study results. As neither the patients nor their physicians were blinded to the change in antipsychotic treatment, potential bias may have been introduced, affecting both patient- and physician-reported subjective outcome measures (such as PANSS and PSP scores, and both patient and physician treatment satisfaction scores).

Although this particular study did not have an oral control group, a recent relapse prevention study showed that in patients recently diagnosed with schizophrenia, treatment with PP1M almost doubled the time to relapse compared with treatment with oral antipsychotics (Schreiner et al. 2015a). The present study provides important information about the potential expectations for treatment outcomes in a clinically representative population (many of whom would not have been eligible for inclusion in RCTs) after switching from atypical oral antipsychotic monotherapy to PP1M in a more real-world environment, and includes clinically relevant insights into dosage selection and adjustments and the use of concomitant medication.

## Conclusions

In conclusion, these data illustrate that non-acute patients (considered clinically stable by their physician) with schizophrenia may show meaningful improvement of psychotic symptoms, functioning, and treatment satisfaction when switched from oral atypical antipsychotic monotherapy to long-acting treatment with PP1M. However, this open-label, naturalistic study did not have an oral control group, limiting the strength of these findings. Validation of these observations in a blinded study with suitable comparator group(s) would be a valuable next step. In this diverse patient population, PP1M was well tolerated, presenting no new safety signals.

## Electronic supplementary material

Supplementary Table 1 (DOCX 13 kb)
