# Supplementary material for: Switching from oral atypical antipsychotic monotherapy to paliperidone palmitate once-monthly in non-acute patients with schizophrenia: A prospective, open-label, interventional study
Source: Psychopharmacology (Berl). 2016 Nov 5;234(1):3–13. doi: 10.1007/s00213-016-4445-0 (PMC5203852; doi:10.1007/s00213-016-4445-0)
Supplement: Supplementary file 1 — (DOCX 13 kb) [file 213_2016_4445_MOESM1_ESM.docx]

**Supplementary Table 1. Weight change following switch to PP1M**

| **Patients switched to PP1M from:** | **ARI** | **OLA** | **Pali ER** | **QUE** | **RIS** |
| --- | --- | --- | --- | --- | --- |
| Weight, n | 43 | 78 | 94 | 41 | 170 |
| Mean baseline weight, kg (SD) | 90.6 (21.9) | 80.9 (16.3) | 80.4 (17.4) | 81.0 (16.4) | 80.3 (17.3) |
| Mean endpoint weight, kg (SD) | 94.1 (23.7) | 80.6 (16.6) | 81.7 (18.1) | 83.2 (16.5) | 81.1 (17.5) |
| **Change from baseline to endpoint, kg (SD)** | **3.5 (6.3)** | **–0.3 (4.6)** | **1.4 (4.3)** | **2.2 (6.1)** | **0.8 (4.6)** |
| 95% CI of mean change | 1.5, 5.4 | –1.3, 0.7 | 0.5, 2.2 | 0.2, 4.1 | 0.1, 1.5 |
| Weight gain ≥7%, n (%) | 12 (27.9) | 8 (10.3) | 14 (14.9) | 11 (26.8) | 20 (11.8) |
| Body mass index, n | 42 | 78 | 94 | 41 | 168 |
| Mean baseline BMI, kg/m^2^ (SD) | 30.4 (7.5) | 27.4 (6.2) | 26.9 (5.5) | 28.2 (5.5) | 27.2 (5.6) |
| Mean endpoint BMI, kg/m^2^ (SD) | 31.6 (8.1) | 27.3 (6.4) | 27.4 (5.8) | 29.1 (6.1) | 27.5 (5.7) |
| **Change from baseline to endpoint, kg/m^2^ (SD)** | **1.2 (2.2)** | **–0.1 (1.6)** | **0.5 (1.5)** | **0.8 (2.3)** | **0.3 (1.6)** |
| 95% CI of mean change | 0.5, 1.9 | –0.5, 0.3 | 0.2, 0.7 | 0.1, 1.6 | 0.1, 0.5 |
| Baseline BMI, kg/m^2^, n (%) |  |  |  |  |  |
| ≤25 | 11 (26.2) | 34 (43.6) | 41 (43.6) | 11 (26.8) | 68 (40.5) |
| 25–30 | 14 (33.3) | 19 (24.4) | 29 (30.9) | 16 (39.0) | 55 (32.7) |
| ≥30 | 17 (40.5) | 25 (32.1) | 24 (25.5) | 14 (34.1) | 45 (26.8) |
| Endpoint BMI, kg/m^2^, n (%) |  |  |  |  |  |
| ≤25 | 7 (16.7) | 32 (41.0) | 37 (39.4) | 8 (19.5) | 53 (31.5) |
| 25–30 | 14 (33.3) | 21 (26.9) | 32 (34.0) | 18 (43.9) | 73 (43.5) |
| ≥30 | 21 (50.0) | 25 (32.1) | 25 (26.6) | 15 (36.6) | 42 (25.0) |

ARI, aripiprazole; BMI, body mass index; CI, confidence interval; OLA, olanzapine; Pali ER, paliperidone extended-release; PP1M, once-monthly paliperidone palmitate; QUE, quetiapine; RIS, risperidone; SD, standard deviation
